# TNF-*α* Induces Neutrophil Apoptosis Delay and Promotes Intestinal Ischemia-Reperfusion-Induced Lung Injury through Activating JNK/FoxO3a Pathway

**DOI:** 10.1155/2021/8302831

**Published:** 2021-12-29

**Authors:** Daili Chen, Chaojin Chen, Xue Xiao, Ziyan Huang, Xiaolei Huang, Weifeng Yao

**Affiliations:** ^1^Department of Anesthesiology, Affiliated Shenzhen Maternity & Child Healthcare Hospital, Southern Medical University, Shenzhen 518028, China; ^2^Department of Anesthesiology, The Third Affiliated Hospital of Sun Yat-sen University, Guangzhou 510630, China

## Abstract

**Background:**

Intestinal ischemia is a common clinical critical illness. Intestinal ischemia-reperfusion (IIR) leads to acute lung injury (ALI), but the causative factors of ALI are unknown. The aim of this study was to reveal the causative factors and mechanisms of IIR-induced lung injury.

**Methods:**

A mouse model of IIR was developed using C57BL/6 mice, followed by detection of lung injury status and plasma levels of inflammatory factors in sham-operated mice and model mice. Some model mice were treated with a tumor necrosis factor-*α* (TNF-*α*) inhibitor lenalidomide (10 mg/kg), followed by observation of lung injury status through hematoxylin and eosin staining and detection of neutrophil infiltration levels through naphthol esterase and Ly6G immunohistochemical staining. Additionally, peripheral blood polymorphonuclear neutrophils (PMNs) were cultured *in vitro* and then stimulated by TNF-*α* to mimic *in vivo* inflammatory stimuli; this TNF-*α* stimulation was also performed on PMNs after knockdown of FoxO3a or treatment with the c-Jun N-terminal kinase (JNK) inhibitor SP600125. PMN apoptosis after stimulation was detected using flow cytometry. Finally, the role of PMN apoptosis in IIR-induced lung injury was evaluated *in vivo* by detecting the ALI status in the model mice administered with ABT-199, a Bcl-2 inhibitor.

**Results:**

IIR led to pulmonary histopathological injury and increased lung water content, which were accompanied by increased plasma levels of inflammatory factors, with the TNF-*α* plasma level showing the most pronounced increase. Inhibition of TNF-*α* led to effective reduction of lung tissue injury, especially that of the damaging infiltration of PMNs in the lung. *In vitro* knockdown of FoxO3a or inhibition of JNK activity could inhibit TNF-*α*-induced PMN apoptosis. Further *in vivo* experiments revealed that ABT-199 effectively alleviated lung injury and decreased inflammation levels by promoting PMN apoptosis during IIR-induced lung injury.

**Conclusion:**

TNF-*α* activates the JNK/FoxO3a pathway to induce a delay in PMN apoptosis, which promotes IIR-induced lung injury.

## 1. Introduction

Intestinal ischemia-reperfusion (IIR) injury is a common clinical phenomenon that mostly occurs during severe trauma, shock, infection, heart failure, and some surgical operations (e.g., acute mesenteric ischemia, small bowel transplantation, abdominal aortic aneurysm surgery, and extracorporeal circulation). IIR can lead to subsequent multiple organ dysfunction syndrome (MODS) or even multiple organ failure (e.g., the lung and the brain). Therefore, the intestine is considered to be the “hub” or the “initiating” organ of MODS, and intestinal injury is an important factor leading to death in critically ill patients [1]. Postoperative acute lung injury (ALI) and acute respiratory distress syndrome (ARDS) are independent risk factors for prolonged intensive care unit (ICU) stay and increased medical costs [2]. The mortality due to disease deterioration among the ALI patients treated in the ICU is still nearly 40% [3]. However, it is still unclear how IIR injury induces lung injury. Recently, Ma et al. showed that reactive oxygen species (ROS) maybe the injurious factor produced from ischemia intestine, and mesenteric lymphatics may serve as an important conduit of gut-lung crosstalk in inflammation [4], to investigate the direct causative factors of ALI caused by IIR, and methods to intervene may promote the early prognosis of patients.

Studies have shown that inflammatory cells play a key role in the development of ALI. Rapid infiltration of large numbers of inflammatory cells, especially polymorphonuclear neutrophils (PMNs), in the lung is an important indication of ALI, while the apoptosis and clearance of PMNs are predictive of inflammation regression [5]. And the clearance of apoptotic neutrophils by macrophages promotes anti-inflammatory signaling. Additionally, the delay of PMN apoptosis may be an important factor responsible for the persistent progression or deterioration of ALI [6, 7]. However, the regulatory mechanism of the PMN apoptosis delay during IIR-induced lung injury is still unknown.

FoxO is a transcription factor that regulates the biological activity of cells by binding to different functional target genes, including those of the regulation of apoptosis, cell cycle, deoxyribonucleic acid (DNA) repair, oxidative stress, cell differentiation, and glucose metabolism [8]. The conserved FoxO subfamily in mammals consists of four members: FoxO1, FoxO3, FoxO4, and FoxO6 [9]. Out of the other FoxO members, FoxO3a plays a major role in apoptosis regulation and leads to cell apoptosis by regulating the expression of cell death receptor ligands such as tumor-necrosis factor-related apoptosis-inducing ligand (TRAIL) [10, 11]. Recently, Hong et al. found that FoxO3a activation takes part in ALI induced by lipopolysaccharide (LPS) through regulating autophagy [12]. However, it remains unclear whether FoxO3a plays a role in PMN apoptosis and is related to IIR-induced lung injury.

The aim of this study was to construct animal models of IIR-induced lung injury and *in vitro* models of PMN-induced inflammatory injury to investigate the causative factors of IIR-induced ALI as well as the process and mechanism through which the causative factors promote the infiltration of PMNs and delay PMN apoptosis.

## 2. Materials and Methodology

### 2.1. Materials

The materials are as follows: recombinant TNF-*α* (PeproTech, NJ, USA); ABT-199, SP600125, and lenalidomide (MedChemExpress, NJ, USA); TNF-*α*, myeloperoxidase (MPO), interleukin-1 *β* (IL-1*β*), IL-6, IL-8, and IL-18 enzyme linked immunosorbent assay (ELISA) kits (BD Biosciences, CA, USA); rabbit anti-mouse FoxO3a antibody, rabbit anti-mouse Ly6G antibody, rabbit anti-mouse JNK antibody, rabbit anti-mouse Bcl-2 antibody, rabbit anti-mouse glyceraldehyde-3-phosphate dehydrogenase (GAPDH) antibody, horseradish peroxidase- (HRP-) labeled goat anti-rabbit secondary antibody (Abcam, Cambridge, UK); HRP-labeled anti-mouse secondary antibody, bicinchoninic acid (BCA) Protein Assay Kit (Beyotime Biotechnology, Shanghai, China); Naphthol AS-D Chloroacetate (Specific Esterase) Kit (Sigma, NY, USA); Diaminobenzidine DAB Kit (ZLI9019; ZSGB-BIO); Dulbecco's modified Eagle's medium (DMEM) medium, trypsin, and fetal bovine serum (FBS) (Gibco, NY, USA); FoxO3a small interfering ribonucleic acid (siRNA) (Guangzhou RiboBio Co., Ltd., Guangzhou, China); and Cell counting kit-8 (CCK-8) and Annexin V-fluorescein isothiocyanate/propidium iodide (FITC/PI) Apoptosis Assay Kit (Jiangsu KeyGEN BioTECH Corp., Ltd., Jiangsu, China).

### 2.2. Animal Grouping

A total of 48 clean-grade and healthy male C57BL/6 mice were provided by the Institute of Zoology, Guangdong Academy of Sciences, each weighing 20–25 g and aged 6–8 weeks, and were maintained at 22 ± 2°C and 50 ± 5% relative humidity with a 12 h light/dark cycle. The 48 male mice were completely randomized into six groups (*n* = 6 each) to receive different treatments as follows: sham surgery, IIR, lenalidomide, IIR+lenalidomide, ABT-199, and IIR+ABT-199. All experiments were approved by the Animal Ethics Committee of South China Agricultural University (2021D028).

### 2.3. Animal Modeling of IIR Injury and Intervention with Drugs

The IIR group mice were modeled by clamping the superior mesenteric artery (SMA) for a certain period of time followed by perfusion. Specifically, C57BL/6 mice were weighed, anesthetized by inhalation of 2% isoflurane, routinely disinfected, and then, subjected to an incision (3-4 cm) in the midline of the abdomen to expose the SMA. The SMA was first clamped at the starting part with a noninvasive arterial clamp for 30 min, and then, the SMA blood flow was restored by removing the clamp, which was followed by sampling after 6 h. The sham group was subjected to the same procedure except for SMA clamping [13–15]. The lenalidomide group was sequentially subjected to sham surgery, intravenous administration of lenalidomide (10 mg/kg), and sampling after 6 h [16]. In the IIR+lenalidomide group, SMA clamping was followed by clamp removal as well as immediate intravenous administration of lenalidomide (10 mg/kg), which was followed by sampling after 6 h. The ABT-199 group was sequentially subjected to sham surgery, intravenous administration of SMA (10 mg/kg), and sampling after 6 h. In the IIR+ABT-199 group, intravenous administration of ABT-199 was also given upon clamp removal after 30 minutes of SMA clamping and was reflowed by sampling after 6 h.

### 2.4. Pathological Testing

The experiment mice were euthanized under deep anesthesia with 8% isoflurane and blood sampling from the abdominal aorta. A piece (2 cm) of the small intestine was dissected and collected at 8 cm from the pylorus and cleaned and fixed with 4% paraformaldehyde. The chest cavity was opened to expose both lungs, and the upper lobe of the right lung was harvested and fixed with 4% paraformaldehyde. The fixed tissue was then embedded with paraffin, sectioned, and stained with hematoxylin and eosin, which was followed by microscopic observation (Leica Microsystems DM500, German) of the intestinal tissue and pulmonary histopathological changes.

### 2.5. Lung Histopathology Scoring

Histopathological changes of the lung were observed with optical microscopy and were scored in terms of the degree of (1) alveolar congestion, hemorrhage, and edema; (2) PMN infiltration and aggregation in the airways or (and) vascular walls; and (3) alveolar septa thickening and hyaline membrane formation [17, 18]. Each lung section was microscopically observed in three fields of view, and the observation in each field was scored 0–3 according to Koksel's method [19], with score 0 indicating a normal state; score 1 indicating a mild interstitial congestion and PMN infiltration; score 2 indicating a perivascular edema, partial destruction of alveolar structures, and moderate PMN infiltration; and score 3 indicating a structural destruction of lung tissue and massive PMN infiltration. Observation and scoring were performed by blinded testers.

### 2.6. Histochemical Staining with Naphthol AS-D Acetate Esterase to Detect PMN Infiltration in Lung Tissue

PMN infiltration in the lung tissue section was detected using the Naphthol AS-D Chloroacetate (Specific Esterase) Kit according to the kit instructions. After staining, 10 fields of view of the lung tissue region were randomly selected under a microscope at 400x magnification to count the number of infiltrating PMNs in the lung tissue.

### 2.7. Determination of Lung Water Content

The middle lobe of the right lung was taken after 6 h of reperfusion, and its surface were cleaned by filter paper. After measuring the wet weight with an electronic balance, the tissue was placed in a drying oven until a constant weight was reached, and then, the dry weight was measured. Lung water content was determined according to the following formula: Water content = (lung wet weight − lung dry weight)/lung wet weight × 100%.

### 2.8. Measurement of Inflammatory Factor Concentration

Blood samples, the broncho-alveolar lavage fluid, and lung tissue homogenates were collected from the mice using a method reported previously [20]. The levels of inflammatory factors TNF-*α*, IL-1*β*, IL-6, MPO, IL-8, and IL-18 were determined using their specific ELISA kits.

### 2.9. Immunohistochemical Staining

Paraffin-embedded sections were deparaffinized with toluene and routinely hydrated, followed by antigen retrieval, a phosphate-buffered saline (PBS) wash in triplicate, and blocking with 5% bovine serum albumin (BSA) for 1 h at room temperature (26°C). The sections were incubated with the primary antibody against Ly6G (1 : 500) overnight at 4°C, followed by PBS washing in triplicate and incubation with the secondary antibody for 1 h at room temperature (26°C) under lightproof conditions. A DAB Kit was used for visualization and costaining with hematoxylin, and the samples were washed and sealed with coverslips. The images were taken under a microscope, and positive cells were analyzed using the Image J software, which counted the number of positive cells in five high-magnification fields of view of the lung tissue of each mouse.

### 2.10. Western Blotting

The total cell protein was extracted, and its concentration was quantified using the BCA protein quantification kit with a loading amount of approximately 30 *μ*g per well. The proteins were separated by sodium dodecyl sulfate-polyacrylamide gel electrophoresis, transferred onto a polyvinylidene diflouride membrane, blocked with 50 g/L BSA for 1 h, and separately incubated with rabbit anti-mouse Bcl-2 antibody (1 : 1,000), rabbit anti-mouse JNK antibody (1 : 100), rabbit anti-mouse FoxO3a antibody (1 : 500), and rabbit anti-mouse GAPDH antibody (1 : 1,000) overnight at 4°C. Next, the incubated membrane was washed with PBS and Tween 20 (PBST) three times and then incubated with HRP-labeled goat anti-rabbit secondary antibody (1 : 2,000) at room temperature (26°C) for 2 h, followed by three PBST washes and enhanced chemiluminescence (ECL) detection. All the Western blotting experimental results were analyzed with Image J.

### 2.11. PMN Isolation and Culture [21]

Human peripheral venous blood was collected from healthy adult volunteers, followed by immediate PMN isolation and culture. Specifically, 3 mL of ethylene diamine tetraacetic acid- (EDTA-) anticoagulated whole blood was gently placed on the surface of 5 mL of PMN isolation medium, and the resulting system was centrifuged at 600× g for 30 min and formed six cell layers in the centrifuge tube, referred to as layers 1 to 6 from top to bottom. Layer 4 that consisted of PMNs was aspirated to another centrifuge tube, followed by addition of 10 mL wash buffer, thorough mixing, centrifugation for 10 min at 300× g, and disposal of the supernatant. Next, erythrocyte lysis solution was added in the centrifuge tube to lyse erythrocytes at 4°C for 10 min, followed by centrifugation for 10 min at 300× g, and disposal of the red supernatant. The precipitated cells were repeatedly resuspended by wash buffer and centrifuged to keep the sediment for 3 times. Finally, the precipitated cells were resuspended in 3 mL of Roswell Park Memorial Institute (RPMI) 1640 culture medium containing 10% FBS and culturing the suspension at 37°C with 95% humidity under 5% CO_2_.

### 2.12. Cell Transfection

Cells reaching 80% confluence were transfected with siRNA-FoxO3a and siRNA-scramble (negative control group) using the LipofectamineTM2000 transfection kit. Specifically, appropriate amounts of siRNA-liposome complexes formed with Lipofectamine™2000 were diluted using serum-free medium and allowed to settle for 5 min and then added into the medium of cultured cells for 48 h of transfection. The primer sequences were synthesized and provided by Guangzhou RiboBio Co., Ltd. Sequences of siRNA-FoxO3a are: sense 5′-GCACAGAGUUGGAUGAAGUTT-3′; antisense 5′-ACUUCAUCCAACUCUGUGCTT-3′. Sequences of scramble siRNA are: sense 5′-UUCUCCGAACGUGUCACGUTT-3′; antisense 5′-ACGUGACACGUU CGGAGAATT-3′.

### 2.13. CCK-8 Detection

The viability of PMNs was detected using the CCK-8 kit according to the kit instructions. Specifically, cells were cultured and intervened in 96-well plates, then 10 *μ*L of the CCK-8 solution was added into each well for 2 h of incubation. The absorbance of each well was measured at 450 nm with a microplate reader. The experimental procedure above was repeated in triplicate.

### 2.14. Apoptosis Detection with Flow Cytometry

The treated cells were collected, washed three times with 1× PBS, and then, resuspended by 800 *μ*L of PBS, followed by sequential addition of 5 *μ*L Annexin V and 5 *μ*L PI. The mixture was allowed to react for 20 min at room temperature under lightproof conditions, followed by flow cytometry to determine the apoptosis rate (AR) according to the formula: AR = number of apoptotic cells/number of total cells × 100%.

### 2.15. Statistical Analysis

All data were expressed as mean ± standard deviation (SD) and processed with statistical software SPSS17.0. Multigroup comparisons were performed using one-way ANOVA, and pairwise group comparisons were performed using the *Tukey* test, with *p* < 0.05 indicating statistically significant differences.

## 3. Results

### 3.1. TNF-*α* Was Identified as an Important Inflammatory Factor Mediating IIR-Induced Remote Lung Injury

**Figure 1:**
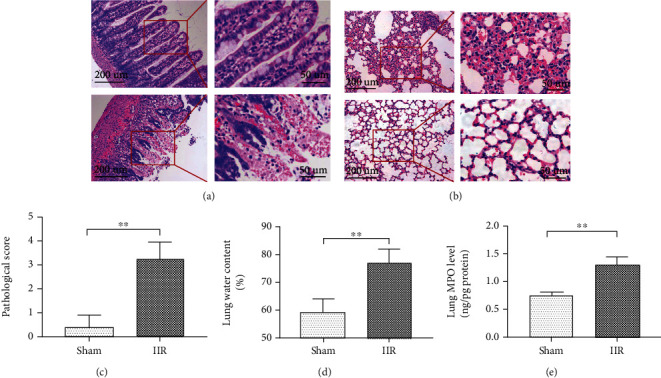
Intestinal ischemia-reperfusion- (IIR-) induced acute lung injury. A mouse model of IIR injury was constructed, and the pathomorphological changes of the small intestinal tissue (a) and lung tissue (b) were detected using hematoxylin and eosin (HE) staining. The extent of lung injury was assessed by Koksel's scoring method (c), and the water content of lung tissue was also determined (d). Changes in the expression of the polymorphonuclear neutrophil (PMN) marker MPO in lung tissue were detected using ELISA. Each bar represents the mean ± SD (*n* = 6 per group). ^∗^*p* < 0.05, ^∗∗^*p* < 0.01, one-way *ANOVA* with Tukey test. Sham: sham-operated group; IIR: intestinal ischemia-reperfusion model group.

In the sham-operated group, the intestinal villi were intact, and there was no subepithelial space expansion. In the IIR group, the top of the intestinal villi epithelium was separated from the lamina propria after reperfusion, concomitant with the exposure, hemorrhage, and ulceration of lamina propria capillaries, along with an increase in the number of inflammatory cells in the lamina propria (Figure 1(a)). The lung tissue structure of the sham-operated group was normal, with a small amount of exudation in the alveolar cavity and no obvious capillary congestion or hemorrhage. By contrast, the IIR group showed significant destruction of the alveolar structure, thickening of the alveolar wall with hemorrhage (Figures 1(b) and 1(c)), edema of the pulmonary interstitium and alveoli (Figure 1(d)), and massive inflammatory cell infiltration. Moreover, the IIR group showed a higher lung water content and elevated expression of MPO, a PMN marker, than the sham-operated group (*p* < 0.01 vs. sham group) (Figure 1(e)).

**Figure 2:**
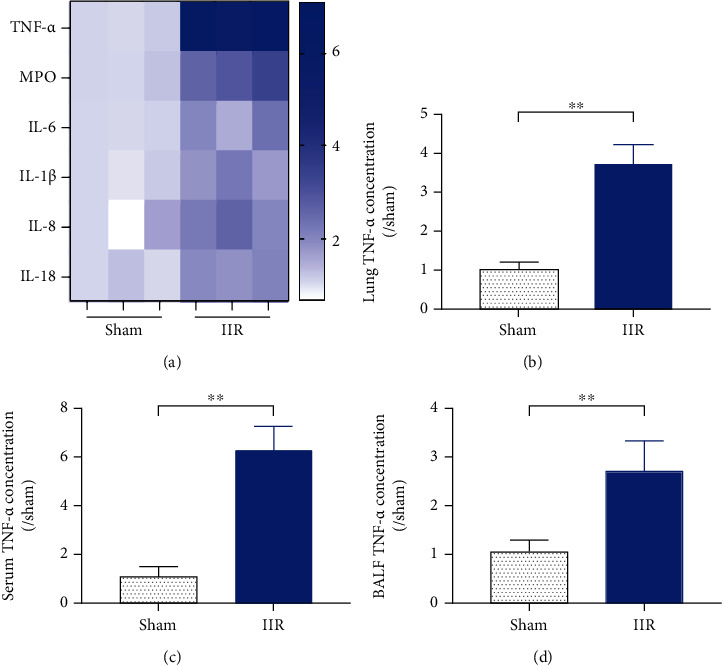
TNF-*α* is identified as an important inflammatory factor mediating intestinal ischemia-reperfusion- (IIR-) induced remote lung injury. Mouse plasma was isolated, and the plasma levels of inflammatory factors TNF-*α*, MPO, IL-1*β*, IL-6, IL-8, and IL-18 were measured using ELISA (a); the levels of TNF-*α* in the lung tissue (b), plasma (c), and alveolar lavage fluid (BALF) (d) were further compared using ELISA. Each bar represents the mean ± SD (*n* = 6 per group). ^∗^*p* < 0.05, ^∗∗^*p* < 0.01, one-way *ANOVA* with Tukey test. Sham: sham-operated group; IIR: intestinal ischemia-reperfusion model group.

To evaluate the main inflammatory factors causing lung injury during IIR, the plasma levels of inflammatory factors TNF-*α*, IL-1*β*, IL-6, IL-8, IL-18, and MPO were determined (Figure 2(a)). TNF-*α* showed significant level changes in the plasma in IIR group (*p* < 0.01 vs. sham group) (Figure 2(b)). Further detection of TNF-*α* levels in the lung tissue (Figure 2(c)) and alveolar lavage fluid (Figure 2(d)) showed that the levels were significantly higher in the IIR group than in the sham-operated group (*p* < 0.01 vs. sham group).

### 3.2. TNF-*α* Inhibitors Effectively Mitigated IIR-Induced Lung Injury by Inhibiting PMN Infiltration

**Figure 3:**
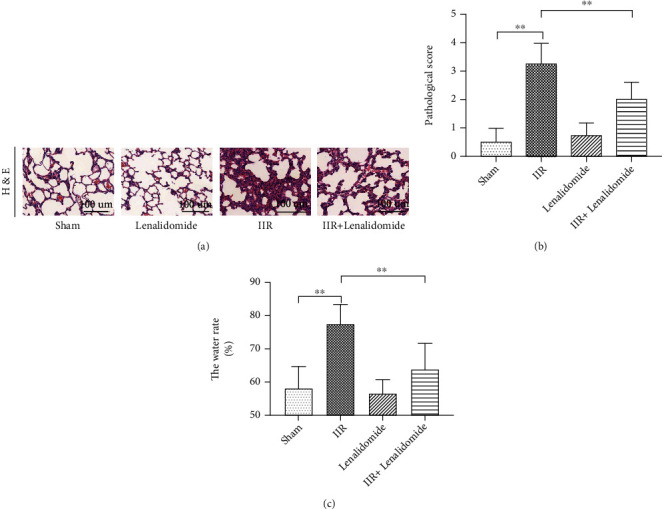
TNF-*α* inhibitors alleviate intestinal ischemia-reperfusion- (IIR-) induced lung injury. A mouse model of IIR injury was constructed and treated with a TNF-*α* inhibitor, followed by using hematoxylin and eosin (HE) staining to detect the pathomorphological changes in the lung tissue (a). The extent of lung injury was assessed using HE staining of lung tissue (b), and the water content of lung tissue was also determined (c). Each bar represents the mean ± SD (*n* = 6 per group). ^∗^*p* < 0.05, ^∗∗^*p* < 0.01, one-way *ANOVA* with Tukey test. Sham: sham-operated group; IIR: intestinal ischemia-reperfusion model group; lenalidomide: group treated with intravenous administration of lenalidomide (10 mg/kg) alone; IIR+lenalidomide group: immediate intravenous administration of lenalidomide (10 mg/kg) at the onset of IIR.

**Figure 4:**
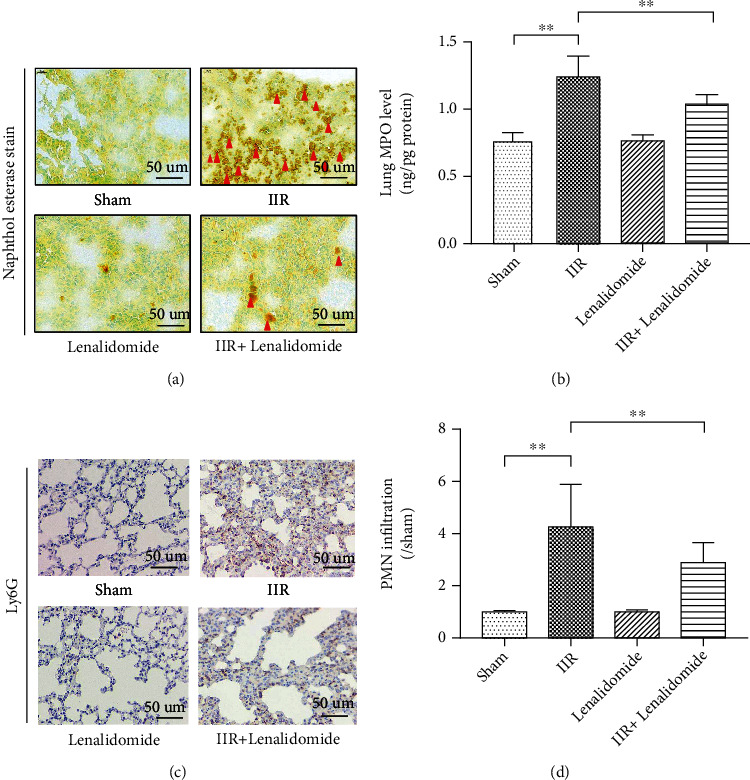
TNF-*α* inhibitor inhibits polymorphonuclear neutrophil (PMN) infiltration of lung tissue. A mouse model of IIR injury was constructed and treated with TNF-*α* inhibitor lenalidomide, followed by using naphthol esterase staining to detect PMN infiltration of lung tissue (a); the expression level of MPO, a PMN marker, in lung tissue was detected using ELISA (b); and the number of Ly6G-positive PMNs in lung tissue was detected (c) and counted (d) using immunohistochemical staining. Each bar represents the mean ± SD (*n* = 6 per group). ^∗^*p* < 0.05, ^∗∗^*p* < 0.01, one-way *ANOVA* with Tukey test. Sham: sham-operated group; IIR: intestinal ischemia-reperfusion model group; lenalidomide: intravenous administration of lenalidomide (10 mg/kg) alone; IIR+lenalidomide: immediate intravenous administration of lenalidomide (10 mg/kg) at the onset of IIR.

To clarify whether TNF-*α* is the main factor of IIR-induced ALI, we treated the IIR model with lenalidomide, a TNF-*α* inhibitor. The results showed that lenalidomide significantly reduced the pathological damage of the lung tissue (Figures 3(a) and 3(b)) and reduced lung tissue edema (*p* < 0.01 vs. IIR group) (Figure 3(c)). Subsequently, it was observed that the IIR group showed significant increase in naphthol esterase-positive PMN infiltration (*p* < 0.01 vs. sham group) (Figure 4(a)), lung MPO expression (Figure 4(b)), and the number of Ly6G-positive PMNs (Figures 4(c) and 4(d)), indicating that PMN infiltration of lung tissue was significant during IIR and that the use of TNF-*α* inhibitor lenalidomide significantly reduced the number of naphthol esterase-positive PMNs and Ly6G-positive PMNs in the lung tissue (*p* < 0.01 vs. IIR group).

### 3.3. *In Vitro* Experiments Confirmed That TNF-*α* Can Affect PMN Apoptosis via FoxO3a

**Figure 5:**
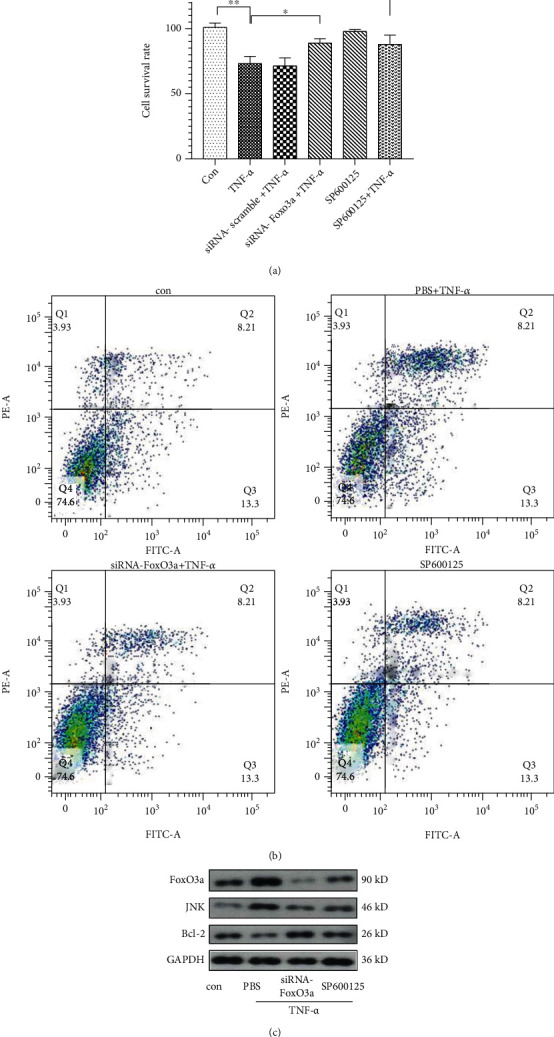
TNF-*α* affects polymorphonuclear neutrophil (PMN) apoptosis through the JNK/FoxO3a pathway. TNF-*α* stimulated PMNs to mimic the in vivo inflammatory environment, and the level of PMN injury was detected using the CCK-8 method (a); the rate of PMN apoptosis was detected using flow cytometry (b); the levels of FoxO3a, JNK, and Bcl-2 were detected using western blotting (c). The data are presented as the mean ± SD, *n* = 6 independent experiments. ^∗^*p* < 0.05, ^∗∗^*p* < 0.01, one-way *ANOVA* with Tukey test. Con: control group; TNF-*α*: a model group in which PMNs were stimulated using TNF-*α* (100 ng/mL) for 12 h; siRNA-scramble+TNF-*α*: the negative control group; siRNA-FoxO3a+TNF-*α*: PMNs treated with TNF-*α* (100 ng/mL) for 12 h at 48 h after transfection with siRNA-FoxO3a; SP600125: PMNs treated with the antiapoptotic agent SP600125 (20 *μ*M) alone; SP600125+TNF-*α*: PMNs treated with TNF-*α* (100 ng/mL) and SP600125 (20 *μ*M) for 12 h; PBS: phosphate-buffered saline.

To further clarify whether TNF-*α* affects the state of PMNs, peripheral blood PMNs were first cultured *in vitro* and then stimulated by TNF-*α* to mimic *in vivo* inflammation. TNF-*α* stimulation can lead to PMN injury, which manifested as a decrease in the number of surviving cells (*p* < 0.01 vs. Con group) (Figure 5(a)) and an increase in the rate of apoptosis (Figure 5(b)). Western blot detection revealed that the levels of FoxO3a and JNK in PMNs were significantly upregulated in TNF-*α* group (Figure 5(c)). Furthermore, FoxO3a siRNA was used to knockdown FoxO3a expression (Supplemental Figure 1). Knockdown of the FoxO3a gene in PMNs followed by TNF-*α* stimulation led to amelioration of PMNs apoptosis and reduced PMN expression of Bcl-2 (Figures 5(b) and 5(c)). To further clarify the role of FoxO3a, the JNK inhibitor SP600125 was applied in PMNs under TNF-*α* stimulation, which could lead to reduce revealed number of apoptotic PMNs (Figure 5(b)), a decrease in the expression of FoxO3a and Bcl-2 (Figure 5(c)).

### 3.4. Promoting PMN Apoptosis during Inflammation Reduced IIR-Induced Lung Injury

**Figure 6:**
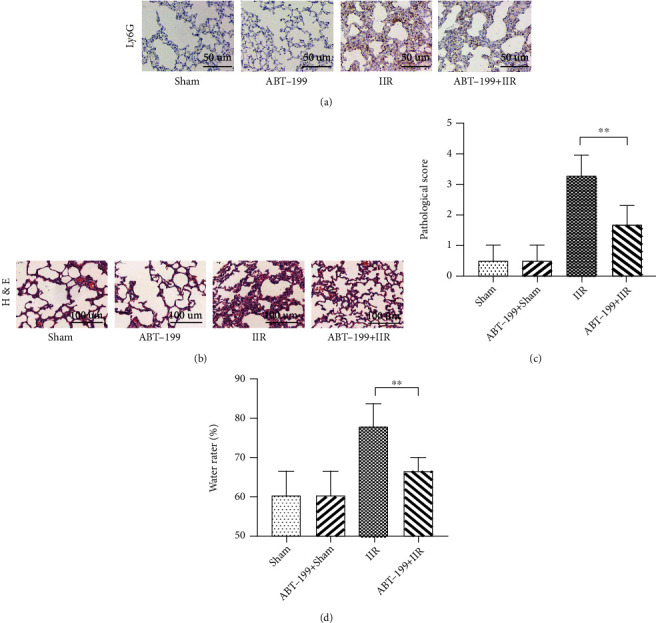
ABT-199 promotes polymorphonuclear neutrophil (PMN) apoptosis to alleviate intestinal ischemia-reperfusion- (IIR-) induced lung injury. A mouse model of IIR injury was constructed and treated with Bcl-2 inhibitor ABT-199, followed by using immunohistochemical staining to detect the infiltration of Ly6G-positive PMNs in the lung tissue (a) and hematoxylin and eosin (HE) staining to detect the pathomorphological changes in the lung tissue (b). The extent of lung injury was assessed by Koksel's scoring method (c), and the water content of lung tissue was also determined (d). Each bar represents the mean ± SD (*n* = 6 per group). ^∗^*p* < 0.05, ^∗∗^*p* < 0.01, one-way *ANOVA* with Tukey test. Sham: sham-operated group; IIR: intestinal ischemia-reperfusion model group; ABT-199: intravenous administration of ABT-199 (10 mg/kg) alone; ABT-199+IIR group: immediate administration of ABT-199 (10 mg/kg) at the onset of IIR.

**Figure 7:**
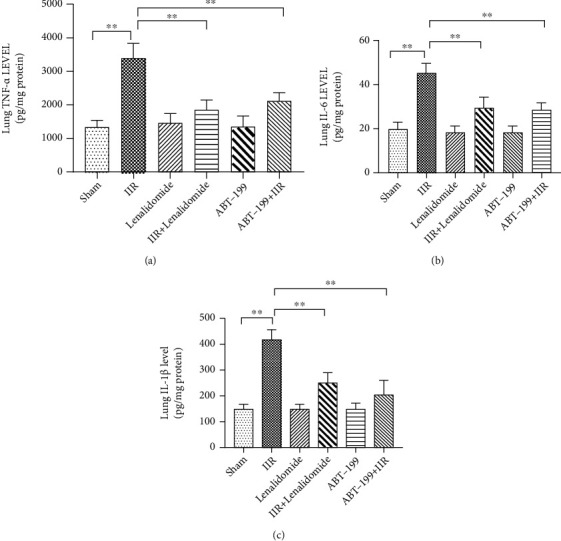
TNF-*α* inhibitor and Bcl-2 inhibitor alleviated the inflammatory response of lung tissue during intestinal ischemia-reperfusion (IIR). A mouse model of IIR injury was constructed and treated with TNF-*α* inhibitor lenalidomide or Bcl-2 inhibitor ABT-199, followed by performing ELISA to detect the levels of inflammatory factors TNF-*α* (a), IL-6 (b), and IL-1*β* (c) in the lung tissue. Each bar represents the mean ± SD (*n* = 6 per group). ^∗^*p* < 0.05, ^∗∗^*p* < 0.01, one-way *ANOVA* with Tukey test. Sham: sham-operated group; IIR: intestinal ischemia-reperfusion model group; ABT-199: intravenous administration of ABT-199 (10 mg/kg) alone; ABT-199+IIR group: immediate administration of ABT-199 (10 mg/kg) at the onset of IIR; lenalidomide: intravenous administration of lenalidomide (10 mg/kg) alone; IIR+lenalidomide group: immediate intravenous administration of lenalidomide (10 mg/kg) at the onset of IIR.

After confirming that TNF-*α* may aggravate IIR-induced lung injury via affecting PMN apoptosis, we further treated the model mice with the Bcl-2 inhibitor ABT-199, immediately at the onset of IIR injury. ABT-199 significantly inhibited the infiltration of PMNs in the lung tissue, which was manifested as a significant reduction in the number of Ly6G-positive PMNs (Figure 6(a)), accompanied by alleviation of pulmonary histopathological injury (Figures 6(b) and 6(c)) and a decrease in lung water content (*p* < 0.01 vs. IIR group) (Figure 6(d)). Further examination indicated that ABT-199 significantly reduced the levels of TNF-*α*, IL-6, and IL-1*β* in the lung tissue (*p* < 0.01 vs. IIR group) (Figures 7(a)–7(c)), suggesting that promoting the apoptosis of PMNs can reduce the level of inflammation in lung tissue and alleviate lung injury in the early stages of lung injury.

## 4. Discussion

A mouse model of IIR-induced lung injury was constructed in this study in order to determine the causative factors of IIR-induced lung injury and the potential mechanisms. The results showed that TNF-*α* was an important causative factor of IIR-induced lung injury, inducing PMN accumulation in the lung tissue and producing severe inflammation, and also caused IIR-induced lung injury via activating the JNK/FoxO3a pathway to regulate PMN apoptosis.

Uncontrolled inflammation is considered to be the early pathogenesis of IIR injury [22–24]. Pulmonary IL-6 promotes inflammation, increases PMN accumulation and infiltration in the lung, and mediates tissue damage, leading to pulmonary edema in the acute stage of ARDS [25]. Research by Park et al. [26] found that the IL-6 concentration in the bronchoalveolar lavage fluid of ARDS and high-risk patients was very high, reaching around 100 times as that found in healthy people, and also, that soluble receptor IL-6 concentration was significantly increased. Mostafa et al. reported [27] that intervention with IL-6 siRNA can reduce the expression of IL-6 in the lung tissue and alleviate lung damage in a mouse model of cecal ligation and puncture (CLP), thereby improving the survival rate of CLP mice. The present study revealed that TNF-*α* was an early-release endogenous inflammatory mediator that played a central role in IIR injury. Macrophage is the main cell type that produces TNF-*α* and interestingly is also highly responsive to TNF-*α* [28]. TNF-*α* may be released from a variety of cells including macrophages, neutrophils, nature killer cells, T/B lymphocytes, and endothelia cells during intestine injury [29]. TNF-*α* not only directly caused lung tissue damage but also mediated secondary pulmonary injuries by inducing endothelial cells to produce IL-1*β*, IL-6, and other inflammatory factors, thereby being responsible for the “cascade effect” of inflammatory factors. Moreover, administration of the TNF-*α* inhibitor effectively reduced IIR-induced ALI. The present study also revealed that soluble TNF-*α* receptors that antagonize TNF-*α* toxicity can significantly reduce intestinal ischemia-induced pulmonary edema and PMN infiltration, thereby improving lung damage, which is consistent with the findings of Sorkine et al. [30].

Various enzymes, reactive oxygen species, and inflammatory mediators released by PMNs are important factors leading to ALI. Therefore, the number of PMNs exuded in the alveoli during ALI, and their subsequent reactions play a key role in ALI development and progression. PMNs have a short survival time in tissues under normal circumstances [31] and are quickly cleared via apoptosis. However, PMN apoptosis in the alveoli is delayed during ALI owing to the antiapoptotic effect of the alveolar exudate and, therefore, the numbers of PMNs and functioning cells increase, which is an important factor for persistent alveolitis during ALI. TNF-*α* enhances the phagocytosis and degranulation functions of PMNs, which cause respiratory bursts and promote their adhesion to endothelial cell- and extracellular matrix protein-coated surfaces, resulting in upregulation of cytokines including IL-1*β* [32]. It is noteworthy that the TNF-*α* inhibitor lenalidomide reduced PMN infiltration in the present study, which may contribute to the TNF-*α*-promoted delay in PMN apoptosis. Consistent with the report from Jonsson et al. [33], which observed that FoxO3a knockout mice showed upregulated Fas ligand expression and exhibited PMN apoptosis under the action of TNF-*α* and IL-1, Fas ligand blockade made FoxO3a-deficient mice prone to arthritis and peritonitis. It was observed in the present study that FoxO3a knockdown PMNs presented with higher cellular survival and upregulated Bcl-2 expression and apoptosis under the action of TNF-*α*, while Bcl-2 inhibition by ABT-199 alleviated ALI in IIR mice.

The PMN apoptosis delay in the alveoli during ALI is closely associated with alveolar exudate, which contains various inflammatory mediators, including IL-1*β*, as an important factor affecting PMN apoptosis. IL-1*β* has been reported to have an antiapoptotic effect on PMNs *in vitro* [34]. It has also been reported that the content of IL-1*β* in the lung increases after the onset of ALI, and PMNs are the main source of IL-1*β* [35]. Results from the present study also showed that the IL-1*β* expression by PMNs in the alveoli increased during ALI and was related to PMN apoptosis delay. IL-1*β* exerts the antiapoptotic effect mainly by inducing the expression of certain Bcl-2 family genes in the PMNs through activation of extracellular signal-regulated kinase [36, 37]. Moreover, IL-1*β* could induce PI3K/AKT phosphorylation which lead to inflammation [38]. Activation of the PI3K/Akt/FoxO3a pathway participated in the process of hyperoxia-induced type II alveolar epithelial cells apoptosis [39]. Taken together, IL-1*β* maybe another cytokine which induce PMN apoptosis delay via activating pathway like PI3K/Akt/FoxO3a.

The present study has the following limitations: (1) although TNF-*α* has been found to be an important factor leading to lung injury in the early stages of IIR, the IIR-induced TNF-*α* release and the specific inflammatory cells that release TNF-*α* are still unclear. (2) Although it has been observed that TNF-*α* regulates PMN apoptosis through the JNK/FoxO3a pathway, it is unclear whether TNF-*α* also affects the activity and phagocytosis of macrophages, considering that PMN apoptosis is closely related to the phagocytosis and clearance capacity of macrophages. (3) Proinflammatory cytokines including granulocyte-macrophage colony-stimulating factor (GM-CSF) and interleukin-8 (IL-8) have been reported to extend the half-life of neutrophil in inflamed site and IL-10 to accelerate the apoptosis of neutrophil [40], whether other cytokines such as IL-1*β*, IL-6, IL-8, IL-18, and MPO whose change have been detected in the damage lung could also affect the process of neutrophil apoptosis is still unclear.

**Figure 8:**
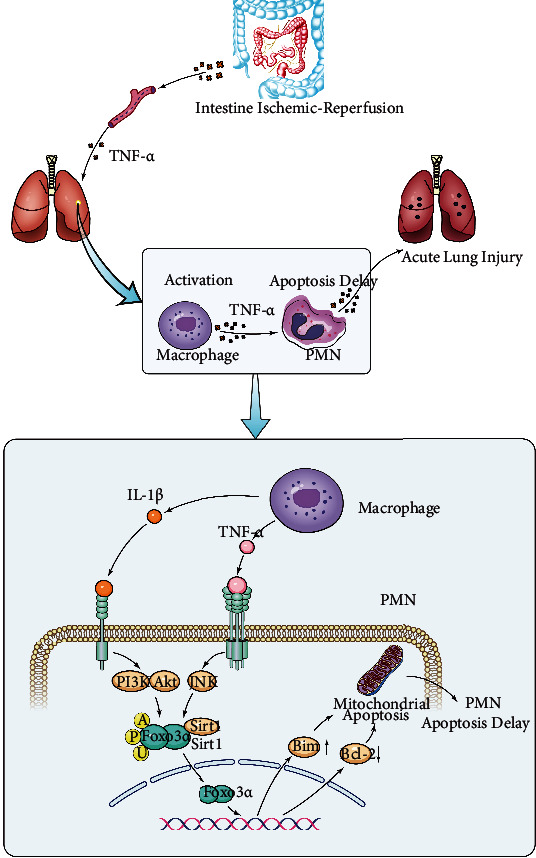
Schematic illustration of the mechanism of TNF-*α*-induced delay in polymorphonuclear neutrophil (PMN) apoptosis that promotes intestinal ischemia-reperfusion- (IIR-) induced lung injury via activating FoxO3a. TNF-*α* releases into the circulation and activates macrophages to produce a large amount of TNF-*α* during intestinal ischemia-reperfusion. TNF-*α* subsequently promotes the dissociation of FoxO3a from Sirt1 into the nucleus by activating the JNK pathway in neutrophil, which promotes the transcription of Bim and inhibits the transcription of Bcl-2, resulting in delayed neutrophil apoptosis and thus induces acute lung injury. Moreover, IL-1*β* released by macrophages may also activate nuclear translocation of FoxO3a through the PI3K/AKT pathway in neutrophil.

In summary, the present study shows that TNF-*α* is an important causative factor of lung injury in the early stages of IIR, and TNF-*α* promotes IIR-induced lung injury by inducing a delay in PMN apoptosis through the JNK/FoxO3a pathway (Figure 8).

## Data Availability

The datasets used and/or analysed during the current study are available from the corresponding author on reasonable request.

## Supplementary Materials

Supplementary Materials Supplemental Figure 1: the effects of FoxO3a siRNA on polymorphonuclear neutrophil (PMN). (a) FoxO3a protein expression level was detected by using western blot method. (b) Analysis of western blot band based on (a).
