# Supplementary material for: TNF-α Induces Neutrophil Apoptosis Delay and Promotes Intestinal Ischemia-Reperfusion-Induced Lung Injury through Activating JNK/FoxO3a Pathway
Source: Oxid Med Cell Longev. 2021 Dec 29;2021:8302831. doi: 10.1155/2021/8302831 (PMC8731283; doi:10.1155/2021/8302831)
Supplement: Supplementary Materials — Supplemental Figure 1: the effects of FoxO3a siRNA on polymorphonuclear neutrophil (PMN). (a) FoxO3a protein expression level was detected by using western blot method. (b) Analysis of western blot band based on (a). [file 8302831.f1.docx]

**TNF-α induces neutrophil apoptosis delay and promotes intestinal ischemia-reperfusion-induced lung injury through activating JNK/FoxO3a pathway**

Daili Chen^1^, Chaojin Chen^2^, Xue Xiao^2^, Ziyan Huang^2^, Xiaolei Huang^1^, Weifeng Yao^2^.

**Supplemental Figure 1**


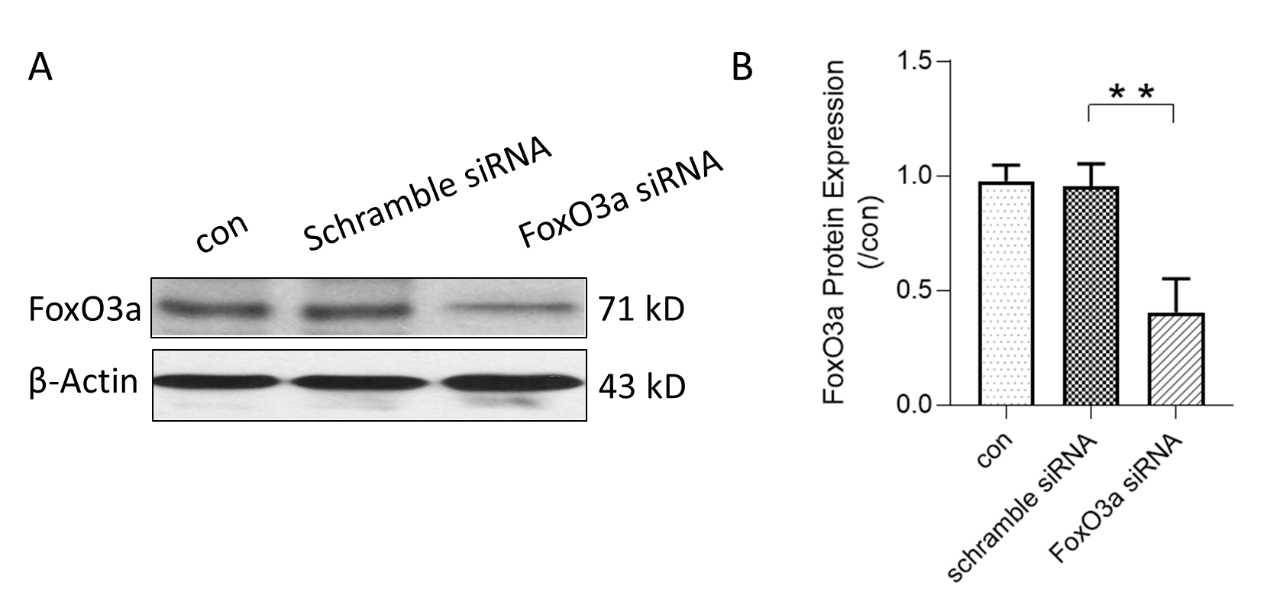


**Supplemental Figure 1: The effects of FoxO3a siRNA on polymorphonuclear neutrophil (PMN).** A, FoxO3a protein expression level was detected by using western blot method. B, Analysis of western blot band based on A.
